# Frequency of lactate elevation following pancreatic surgery and its relationship to postoperative complications

**DOI:** 10.1016/j.sipas.2025.100298

**Published:** 2025-07-24

**Authors:** Lana Othman Mahmmud, Vilhelmas Bartusevicius, Åke Norberg, Poya Ghorbani, Jonathan Grip

**Affiliations:** a Dept. of Clinical Science, Intervention and Technology, CLINTEC, Karolinska Institute, Stockholm, Sweden; b Function Perioperative Medicine and Intensive Care, Karolinska University Hospital, Stockholm Sweden; c Department of Upper Abdominal Diseases, Karolinska University Hospital, Stockholm, Sweden

**Keywords:** Lactate, Pancreatic surgery, Perioperative, Postoperative

## Abstract

**Background:**

Lactate is often elevated following major pancreatic surgery but the clinical relevance of this it not known.

**Methods:**

A retrospective study including 491 consecutive patients undergoing major pancreatic surgery. Lactate upon arrival to post anaesthesia unit (L_0_), the morning following surgery (L_POD1_) and the highest value within those two time points (L_High_) were examined. The primary outcome was postoperative complications (Clavien-Dindo IIIa-V) and the secondary outcomes were surgery specific complications and hospital length of stay.

**Results:**

Median lactate values were: L01.7 mmol/L (IQR: 1.2 –2.6), LPOD11.3 mmol/L (IQR: 0.9 –1.9) and L_High_ 2.3 mmol/L (IQR 1.7 –3.1). There were no differences in lactate values at any measuring point between those developing complications and those that didn´t. AUROC analysis (0.531–0.581) and Youden´s index (0.08–0.17) indicated poor diagnostic performance. L_High_ > 2.65 mmol/L was associated with Odds ratio 2.05 (1.34 –3.14) for developing postoperative complications. Plasma lactate was higher following total pancreatectomy compared to partial resection at all three time points.

**Discussion:**

Plasma lactate elevation in common following pancreatic surgery; however, this is of limited clinical use to predict complications. The relatively higher lactate following total pancreatectomy might be due to hormonal deficits inherent to this procedure.

## Graphical abstract

**Figure:**
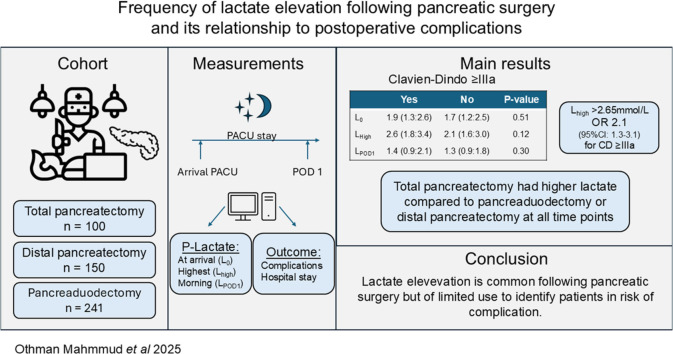

## Introduction

Pancreatic resections are major, complex surgeries that put large physiological stress on the patient. As a result, it is linked to challenging postoperative recovery and high incidence of complications, with rates of 30–40 % [[1], [2], [3]]. Despite various types of pre-, peri‑ and post-surgery optimizations such as, centralization of pancreatic surgery [4], strict fluid management [5], minimally invasive approach [6], different anastomotic techniques [7], and regular blood sampling [8] the morbidity remains high.

Inadequate tissue perfusion can lead to impaired microvascular flow and organ dysfunction, which are major contributors to postoperative complications. Jhanji et al. found that microvascular flow abnormalities were more commonly observed in patients who developed complications following major abdominal surgery [9]. Lactate levels can be used as a marker for tissue hypoperfusion, inadequate tissue oxygenations and as a marker of stress [10,11]. Lactate levels have been recognized as a mean to evaluate the severity of critically ill patients [12]. Elevated lactate levels are also associated with poorer outcomes, both in regards to mortality and morbidity in critically ill patients [13]. It has therefore been hypothesized that elevated plasma lactate can be used to identify patients at risk of developing postoperative complications.

Several single-center studies report significant associations between postoperative complications after major abdominal surgery and plasma lactate levels during the first 24 h after surgery [[14], [15], [16]]. These are however investigating mixed abdominal surgeries, and as some types have higher frequencies of both lactate elevation and complications, it would be more clinically useful to study them separately.

We performed a retrospective analysis to investigate whether lactate levels at arrival to postoperative care unit (PACU) or the morning following surgery showed any correlation to postoperative complications following major pancreatic surgery. We also wanted to explore if there was a threshold for lactate levels during the first night following surgery that could predict complications by examining the highest lactate value during the first night following surgery.

## Methods

### Study population and ethical considerations

This was a single centre retrospective study. The study cohort was all adult patients who underwent pancreatoduodenectomy, distal or total pancreatectomy during a three-year period from January 2016 to December 2018 at the Karolinska University Hospital in Stockholm, a tertiary regional centre for pancreatic surgery. All patients were treated within an Enhanced Recovery Program (ERP), aimed to decrease complications and hospital stay. The concept includes carbohydrate drinks two hours before surgery, strict fluid management, epidural analgesia (EDA), early mobilization and frequent evaluation of arterial blood gas sampling. All patients spend the first night following surgery in a high-dependency postoperative care unit (PACU), with specialised nursing staff and around the clock access to an attending anaesthesiologist. All patients received a buffered dextrose solution (glucose 25mg/ml) at 1 ml/kg/h during the first night. Additionally, Ringers-Acetate was given at the discretion of attending anaesthesiologist. Patient treatment aimed at maintaining a diuresis >0.5 ml/kg/h and mean arterial pressure > 65 mmHg, but could be set higher at the discretion of attending anaesthesiologist in presence of cardiovascular disease. The study was approved by Swedish Ethical Review Authority (Dnr: 2019–04,248) and informed consent was waived. The study was registered at clinicaltrials.gov (NCT06589102).

### Data collection

**Fig. 1:**
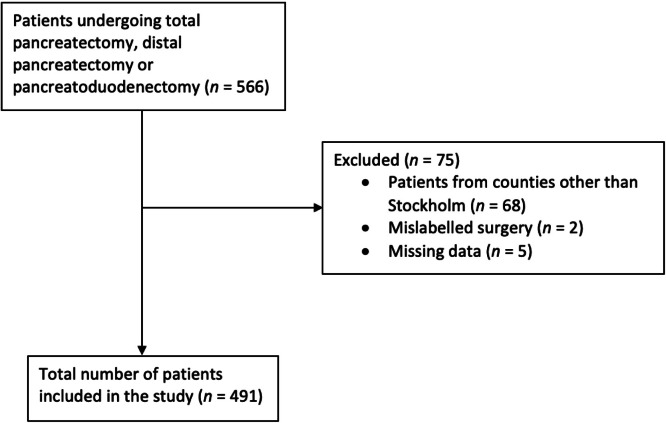
Study inclusion chart. Patients undergoing pancreatic surgery at Karolinska University Hospital 2016–2018 and reasons for exclusion from analysis.

Data on the study population was retrieved from institutional databases. Electronic patient charts (Take Care, ProfDoc, Stockholm, Sweden) were manually scanned for additional information on outcomes and laboratory data were retrieved through central data storage (KarDa, Karolinska University Hospital). Patients with mislabelled surgeries, missing data or residing outside of Stockholm region (due to lost in follow up) were excluded (Fig. 1).

Lactate values at three time points were extracted for each patient, one taken at arrival to the postoperative ward (L_0_), lactate value the following morning, between 6:00 –8:00 (L_POD1_) and the highest lactate value (L_High_) within those two time points. Lactate values were analysed by point-of-care analyser (ABL 800 flex, Radiometer Medical Aps, Copenhagen, Denmark).

### Outcome and data analysis

Priamary outcome was complications according to Clavien-Dindo classification (≥ IIIa) [17] of which ICU admission (CD IV) and need for additional surgery (CD IIIb) were analysed separately as well. Pancreatic surgery specific complication; post operative pancreatic fistula (POPF) and delayed gastric emptying (DGE) defined according to International Study Group of Pancreatic Surgery (ISGPS) [18,19] and bile leakage (BL) according to International Study Group of Liver Surgery 2011 [20] were also examined. Outcomes for POPF, DGE and BL were dichotomized (none and grade A vs. grade B & C), as only grades B and C were deemed as clinically relevant. Kendall’s rank correlation tau (τ) was used to analyse correlation between length of hospital stay following surgery and lactate values at the three time points.

To investigate what factors influenced postoperative lactate values a univariate regression of surgery time, perioperative bleeding, difference in pre- and postoperative weight (as a surrogate for cumulative fluid balance) was performed. Factors that were significant in univariate analysis were included in a multivariate analysis. Binary factors; sex, American Society of Anesthesiologists (ASA)-class (1–2 vs. 3–4), Charlson Comorbidity Index (CCI) Score (0–2 vs. ≥3) and metformin (Glucophage) intake on a regular basis prior to surgery, were analysed through Man-Whitney u-test. A mixed liner model, using all 2604 collected lactate values were used to examine temporal differences between patients with and without complications. A p-value < 0.05 was considered significant and for secondary analyses a Benjamini-Hochberg adjustment was performed to compensate for multiple testing to minimize risk of false positive findings. Data was analysed using R-programming (RStudio, 2023.09.1 + 194 and 2024.12.0 + 467).

## Results

**Fig. 2:**
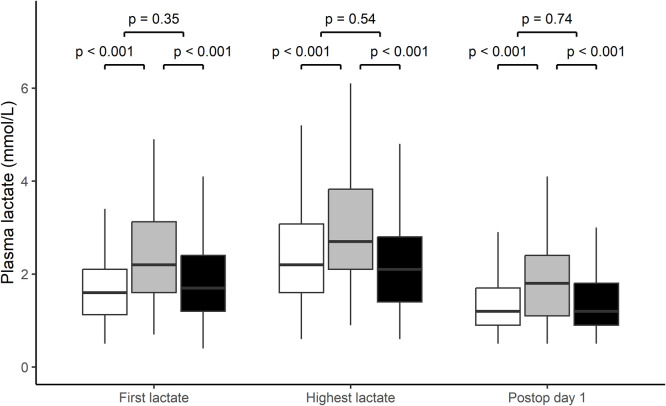
Comparison of plasma lactate at arrival to postoperative care unit (first panel), highest measured plasma lactate between arrival to postoperative care unit and the following morning (middle panel) and the morning following surgery (last panel) between distal pancreatectomy (white), total pancreatectomy (grey) and pancreatoduodenectomy (black) analysed trough Kruskall-Wallis with subsequent pairwise Mann-Whitney U test with Bonferroni correction. Data is presented as median with interquartile ranges, range and outliers.

A total of 491 patient (51 % women) were included in the analysis (Fig. 1). Of these 241 patients underwent pancreatoduodenectomy, 150 distal pancreatectomy and 100 total pancreatectomy (Table 1). Patients had an average of 5.6 lactate measurements in the study period (range 2–15) with no difference between those suffering complications and those that didn´t. Median lactate (IQR) at arrival to PACU was 1.7 (1.2 –2.6) mmol/L, the following morning 1.3 (0.9 –1.9) mmol/L and peak lactate 2.3 (1.7 –3.1) mmol/L. Patients undergoing total pancreatectomy had higher lactate at all three time points, as compared to distal pancreatectomy and pancreatoduodenectomy (Fig. 2). A total of seven patient died before being discharged, two within one week and five after prolonged care for complication.

**Table 1:** Characteristics of 491 patients undergoing pancreatectomy.

	Patients (*n* = 491)
**Female sex**	251 (51.1 %)
**Age**	70 [21,89]
**Body mass index**	25 [17,47]
**Diabetes mellitus**	97 (19.8 %)
**ASA class I-II**	283 (57.6 %)
**ASA-class III-IV**	208 (42.4 %)
**CCI**	
0–2	329 (67.0 %)
3–4	85 (17.3 %)
≥5	77 (15.7 %)
**Diagnosis**	
Pancreatic ductal adenocarcinom	181 (36.9 %)
Intraductal papillary neoplasm	95 (19.3 %)
Chronic pancreatitis	29 (5.9 %)
Other	186 (37.9 %)
**Surgical procedure**	
Distal pancreatectomy	150 (30.5 %)
Total pancreatectomy	100 (20.4 %)
Pancreatoduodenectomy	241 (49.1 %)

ASA –American Society of Anesthesiology. BMI –Body mass index. CCI –Charlson Comorbidity Index. Data is presented as median [range] or number (percentage).

**Fig. 3:**
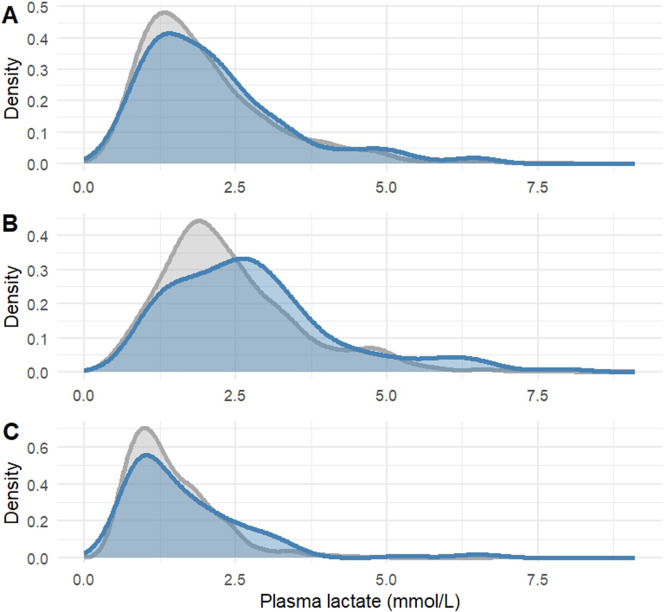
Distribution of plasma lactate measurements for 491 patients undergoing pancreatic surgery presented for patients with postoperative complications (Clavien-Dindo ≥IIIa, blue line, *n* = 116) or without postoperative complications (gray line, *n* = 375). Panel A) at arrival to post anaesthesia care unit (PACU); B) the highest value during treatment in PACU; C) the morning following surgery.

The primary outcome, complication requiring intervention (Clavien-Dindo ≥ IIIa), developed in 116 (24 %) patients. There was no difference between those developing complications and those that did not at any of the time points when results were adjusted for multiple testing (Table 2). A total of 23 patients (4.7 %) were admitted to the ICU following surgery, with no difference in lactate between groups at any time point. This was also true for patients undergoing additional surgery (*n* = 50, 10 %). Bile leakage was developed in 54 patients (11 %) and 164 patients (33 %) developed DGE. POPF developed in 96 patients (24 %) out of 391 patients who underwent pancreatoduodenectomy or distal pancreatectomy. Similar findings (small differences that became statistically non-significant after adjusting for multiple testing) was found for surgery specific complications delayed gastric emptying (at L_0_ and L_POD1_) and bile leakage (at L_0_ and L_High_) but in all other cases there were no difference between groups neither at single examination testing nor after adjusting for multiple tests (Table 2). Lactate had poor diagnostic performance to predict complications (CD ≥ IIIa) in AUROC analysis (0.531–0.581, figure S1) and Youden’s Index (0.08–0.17), but there was a modestly increased risk to develop complications above the thresholds for L_High_ suggested by the Youden´s Index expressed as odds ratio (table S3). The positive predictive value for complications above that threshold was 32 % (55/177 patient with L_High_ developed complications). Density plots for lactate related to complications are presented in Fig. 3. Mixed linear model did not indicate any temporal differences between those that developed complications and those that did not when all lactate values from the first night were included (figure S2). At arrival to PACU plasma lactate showed weak positive correlation to length of stay the morning following surgery τ = 0.1, and a very weak to length of stay directly following surgery, τ = 0.06, and at the highest value, τ = 0.07.

**Table 2:** Plasma lactate concentrations at different time points between those developing complications following pancreatectomy and those that did not.

	L_0_	L_High_	L_POD1_
**CD ≥IIIa** (*n* = 116)	1.9 (1.3, 2.6)	2.6 (1.8, 3.4)	1.4 (0.9, 2.1)
**No CD ≥IIIa** (*n* = 375)	1.7 (1.2, 2.5)	2.1 (1.6, 3.0)	1.3 (0.9, 1.8)
p-value	0.51	0.12	0.30
**ICU stay** (*n* = 23)	1.8 (1.1, 2.3)	2.6 (1.8, 2.9)	1.2 (0.9, 2.0)
**No ICU** (*n* = 468)	1.7 (1.2, 2.6)	2.2 (1.7, 3.1)	1.3 (0.9, 1.9)
p-value	0.73	0.83	0.97
**Additional surgery** (*n* = 50)	1.8 (1.2, 2.4)	2.4 (1.6, 3.1)	1.2 (0.9, 2.2)
**No surgery** (*n* = 441)	1.7 (1.3, 2.6)	2.3 (1.7, 3.1)	1.3 (0.9, 1.8)
p-value	0.82	0.90	0.83
**DGE** (*n* = 165)	2.0 (1.4, 2.7)	2.4 (1.7, 3.2)	1.4 (1.0, 2.1)
**No DGE** (*n* = 325)	1.7 (1.2, 2.4)	2.2 (1.6, 3.0)	1.2 (0.9, 1.8)
p-value	0.13	0.51	0.13
**Bile leakage** (*n* = 54)	2.2 (1.5, 2.8)	2.6 (2.0, 3.2)	1.4 (1.2, 2.0)
**No Bile leakage** (*n* = 434)	1.7 (1.2, 2.4)	2.2 (1.6, 3.0)	1.2 (0.9, 1.8)
p-value	0.13	0.12	0.27
**POPF^a^** (*n* = 96)	1.8 (1.2, 2.4)	2.4 (1.9, 3.2)	1.3 (1.0, 1.8)
**No POPF^a^** (*n* = 291)	1.6 (1.2, 2.4)	2.1 (1.6, 3.0)	1.2 (0.9, 1.7)
p-value	0.56	0.28	0.51

All lactate values are presented in mmol/L. L_0_ - lactate measured at arrival to PACU; L_High_ highest lactate value during stay at PACU; L_POD1_ - lactate value morning following surgery; Plasma lactate are shown as median mmol (IQR-interquartile range); Bile leakage according to ISGLS 2011; DGE - Delayed gastric emptying according to ISGPS 2007; POPF - Postoperative pancreatic fistula according to ISGPS 2007 (Yes = Grade B or C) ^a)^ Only distal pancreatectomy and pancreatoduodenectomy (*n* = 391) were analyzed;.

P-values are assessed with Mann-Whitney U test with Benjamini-Hochberg correction for multiple testing.

Multivariate analysis of continuous variables (perioperative bleeding, duration of operation and leukocyte count) exhibited a weak correlation to L_0_, L_POD1_ and L_High_ (table S1). The univariate analysis of patient related factors, sex, metformin (Glucophage) use, CCI and ASA-class showed no correlation to lactate at any timepoint (Table S2).

## Discussion

The primary aim of this study was to investigate whether plasma lactate levels on the first postoperative day were associated with complications following pancreatic surgery. Our findings indicate that lactate elevation was common in this setting but exhibited no meaningful ability to discriminate between patients who did and did not develop complications, as evidenced by low AUROC values (0.531–0.581) and poor Youden’s index (0.08–0.17). There was an observed odds ratio of 2.05 (95 % CI: 1.34–3.14) for complications in patients with L_High_ >2.65 mmol/L. However, this statistical association does not translate into clinical utility: more patients with L_High_ >2.65 mmol/L remained free of complications than developed them (120 vs. 57) resulting in a positive predictive value of only 32 %. Taken together, these findings suggest that early postoperative lactate measurements provide limited value in identifying patients at risk of complications or prolonged hospitalization following pancreatic surgery in our cohort.

This stands in contrast to studies on mixed abdominal surgery where an association between postoperative lactate and complications have been shown [14–16]. This might however be caused by the fact that certain types of surgeries are associated with lactate increase, while also being more prone to complications, e.g. pancreatic surgery[15]. The reason for the discrepancies is beyond the scope of this study but pancreatic surgery is often associated with local and systemic inflammation, which may play a role in development of both complications and lactate elevation. Pecorelli et al. performed a single-center retrospective study in 1486 patients who underwent elective pancreatic resection between 2015–2019, [22]. 4.4 % of these had unplanned admissions to the ICU following surgery, and increased plasma lactate at the end of surgery (mean of 2.0 vs. 1.2 mmol/L) (OR 1.25, *p* = 0.017) was as an independent factor associated to ICU admission. Although similar frequency of ICU admissions but smaller cohort, we could not replicate this finding. A retrospective study on 156 patients who underwent pancreatic resection and was admitted to ICU for recovery following surgery, found lactate level on arrival to the ICU statistically significant between patients with intra-abdominal infection and those without (3.0 vs. 1.7 mmol/L, *p* < 0.001) [23]. There is no obvious explanation for the difference in our results and other groups. Differences between centers may include patient comorbidities, surgical technique as well as staffing during the *peri* and postoperative care or publication bias.

Postoperative pancreatic fistula is a concerning complication of pancreatic resection, including both distal pancreatectomy and pancreatoduodenectomy. The prediction of POPF continues to be a topic of interest. Our study did not identify any difference in lactate between those that developed POPF and those who did not. This aligns with the findings of Kinaci et al., who studied a smaller group of pancreatoduodenectomy patients (*n* = 85) and reported similar results [24]. Contrarily, Sakamoto et al. found that higher lactate levels could serve as predictors for POPF development [21]. Notably, Kinaci et al. and Sakamoto et al. only included patients who underwent pancreatoduodenectomy, while our study also included distal pancreatectomy patients, resulting in a larger and more heterogeneous cohort.

Lactate metabolism is complex and influenced by several factors, such as underlying comorbidities, pharmacological treatments, perioperative management and surgical stress. This might dilute the stress induced lactate release that could be attributed to complications. Another reason we do not see any difference between the groups might be that the complications arise later, and the sampling time (within 16–18 h) is not sufficient to detect these processes.

The most significant determinant of postoperative lactate was type of surgery, where patients undergoing total pancreatomy had higher lactate levels at all measuring points. This is a novel finding and at first glance somewhat surprising as pancreatoduodenectomy is associated with a larger inflammatory response [25]. The disparity might appear because of a complete loss of pancreatic function, and reduction in glucagon levels. Glucagon deficiency decreases hepatic glucose production, consequently increases levels of gluconeogenetic precursors, lactate, alanine, and pyruvate [26]. The total pancreatomy patients had more bleeding and longer surgeries than the other groups. Indeed, univariate analysis showed that these factors correlated weakly to lactate but seem implausible to account for the entire difference between the groups. When all variables that were significant in univariate analysis were combined (bleeding, duration of surgery and leukocyte) in a multivariate analysis the r^2^ value was still below 0.1 (Table S1).

Our study is subject to limitations. The retrospective and single-centre design may limit the accuracy and generalizability of the findings. Our use of dextrose infusion and sympathetic blockade through epidural analgesia might for example dampen the lactate mobilization. The size of our cohort is large compared to other studies on the same subject and only includes pancreatic surgery. Additionally, the study has few missing data, thereby strengthening the reliability of our results. Initially we were unsure if we could retrieve complete data on postoperative complications and planned to analyse length of stay as a surrogate for this, but as we have complete data on postoperative complications we used this as our primary outcome. Hopefully the physiological lactate response to surgery and treatment can be further explored in future prospective studies.

In conclusion, there is an observed increase in plasma lactate in most patients immediately following pancreatic surgery. This is most likely caused by surgical stress and inflammation rather than early signs of complications. In our setting it was not useful for identifying patients in risk of complications, but hopefully future research will give insights into better monitoring and biomarkers in the immediate postoperative settings. One such example could be micro-dialysis and studies on this is currently ongoing [27].

Patients undergoing total pancreatomy have higher plasma lactate than those undergoing pancreatic resections, possibly caused by alterations of metabolism in the absence of glucagon.

## Statements and declarations

The data that support the findings of this study are available on request from the corresponding author. The data is not publicly available due to privacy or ethical restrictions.

## CRediT authorship contribution statement

**Lana Othman Mahmmud:** Writing – review & editing, Writing – original draft, Visualization, Formal analysis, Data curation. **Vilhelmas Bartusevicius:** Writing – review & editing, Investigation, Formal analysis, Data curation. **Åke Norberg:** Writing – review & editing, Visualization, Software, Methodology, Formal analysis, Data curation. **Poya Ghorbani:** Writing – review & editing, Validation, Resources, Methodology, Formal analysis, Data curation, Conceptualization. **Jonathan Grip:** Writing – review & editing, Writing – original draft, Supervision, Resources, Methodology, Formal analysis, Conceptualization.

## Declaration of competing interest

The authors declare that they have no known competing financial interests or personal relationships that could have appeared to influence the work reported in this paper.

## Appendix Supplementary materials

Image, application 1
