# Supplementary material for: Frequency of lactate elevation following pancreatic surgery and its relationship to postoperative complications
Source: Surg Pract Sci. 2025 Jul 24;22:100298. doi: 10.1016/j.sipas.2025.100298 (PMC12336680; doi:10.1016/j.sipas.2025.100298)

**Supplementary material**

**Table S1.**

Caption: Squared Pearson's correlations coefficients (r^2^) obtained by univariate or multivariate linear regression between log transformed continuous variables and log transformed plasma lactate

| **Univariate analysis** | **Median IQR** | **L_0_ r^2^** | **L_POD1_ r^2^** | **L_High_ r^2^** |
| --- | --- | --- | --- | --- |
| Blood loss (ml) (n=444) | 200 (100-300) | 0.034 *** | 0.040 *** | 0.041 *** |
| Operative time (h) | 6.25 (4.5-7.58) | 0.060 *** | 0.050 *** | 0.030 *** |
| Age (years) | 70 (60-75) | 0.006 | 0.023 *** | < 0.001 |
| WBC (*10^9^/L) | 13.2 (10.9-16.0) | 0.010 * | 0.010 * | 0.026 *** |
| Plasma Amylase (µkat/L) | 0.51 (<0.13-1.28) | < 0.001 | 0.005 | 0.001 |
| Excess body weight (kg) ^a^ (n=462) | 3 (1-4) | 0.001 | 0.010 * | 0.001 |
| **Multivariate analysis** |  |  |  |  |
| Blood loss and OR time |  | 0.071 *** | 0.067 *** | 0.053 *** |
| Blood loss, OR time, and WBC |  | 0.090 *** | 0.087 *** | 0.088 *** |
| Bleeding, OR time, age, and WBC |  |  | 0.099*** |  |

IQR = interquartile range; L_0_ = plasma lactate directly after end of pancreatic surgery; L_POD1_ = plasma lactate at 06:00 the first postoperative day; L_High_ = highest plasma lactate between surgery and the following morning; WBC=white blood cell; OR = operative r^2^ is the squared Pearson's correlation coefficient that tells how much of the dependent variable that can be explained by the independent variable or variables. In this data set r^2^ had to reach at least .008 to be statistically significant. Parameters with p-values under 0.10 were included in a stepwise forward multivariate analysis without the interaction factors between independent variables.
^a^ Fluid load is the difference between the preoperative patient weight and weight at the first postoperative day.
*p < .05; ***p < .001.

**Table S2.**

Caption: Lactate concentrations between dichotomous groups of patients following pancreatic surgery

|  | **L_0_** | **L_High_** | **L_POD1_** |
| --- | --- | --- | --- |
| **Male** (n=240) | 1.8 (1.3, 2.6) | 2.3 (1.7, 3.1) | 1.3 (0.9,1.8) |
| **Female** (n=251) | 1.7 (1.2, 2.5) | 2.2 (1.6-3.0) | 1.2 (0.9, 1.9) |
| p-value | 0.43 | 0.67 | 0.63 |
|  |  |  |  |
| **Metformin** (n=57) | 1.6 (1.1, 2.3) | 2.1 (1.6, 2.8) | 1.3 (0.9, 2.0) |
| **No metformin** (n=434) | 1.8 (1.3, 2.6) | 2.3 (1.7, 3.1) | 1.3 (0.9, 1.9) |
| p-value | 0.43 | 0.67 | 0.67 |
|  |  |  |  |
| **CCI 0-2** (n=329) | 1.7 (1.2, 2.6) | 2.3 (1.7, 3.2) | 1.2 (0.9, 1.8) |
| **CCI ≥ 3** (n=143) | 1.8 (1.3, 2.5) | 2.3 (1.7, 2.9) | 1.5 (1.0, 1.9) |
| p-value | 0.67 | 0.67 | 0.06 |
|  |  |  |  |
| **ASA class 1-2** (n=283) | 1.8 (1.3, 2.7) | 2.3 (1.7, 3.2) | 1.2 (0.9, 1.8) |
| **ASA class 3-4** (n=208) | 1.7 (1.2, 2.3) | 2.2 (1.7, 3.0) | 1.3 (0.9, |
| p-value | 0.34 | 0.67 | 0.67 |
| All lactate values are presented in median concentration in mmol/L with interquartile range. L_0_ - lactate measured at arrival to PACU; L_High_ highest lactate value during stay at PACU; L_POD1_ - lactate value morning following surgery; CCI = Charlson Comorbidity Index; ASA = American Society of Anesthesiologists  P-values are assessed with Mann-Whitney U test with Benjamini-Hochberg correction for multiple testing | | | |

**Table S3.**

Caption: Prognostic performance of plasma lactate to predict complications (defined as Clavien-Dindo ≥ IIIa) following pancreatic surgery

|  | AUROC | J | Optimal cut-off | Odds ratio (95% CI) | p-value |
| --- | --- | --- | --- | --- | --- |
| L_0_ | 0.531 | 0.08 | 1.85 mmol/L | 1.38 (0.91 – 2.10) | 0.387 |
| L_High_ | 0.581 | 0.17 | 2.65 mmol/L | 2.05 (1.34 – 3.14) | 0.021 |
| L_POD1_ | 0.546 | 0.10 | 1.95 mmol/L | 1.76 (1.09 – 2.80) | 0.056 |
| AUROC – area under receiver operating curve; J=Sensitivitet+Specificitet−1 (Youden´s index); Optimal cut-off for lactate value to predict complications according to Youden´s index; odds ratio with 95% confidence intervals for having lactate above cut-off at that measurement; p-value for odds ratio after Bonferroni correction | | | | | |

**Table S4**

Caption: Differences between patients undergoing different types of pancreatic surgery.

|  | **Distal resection** | **Total resection** | **Pancreaduodenoctomy** |
| --- | --- | --- | --- |
| **BMI** | 25 (23, 29) | 26 (23, 29) | 24 (22, 27) |
| **Age** (y) | 69 (62, 74) | 68 (57, 75) | 71 (62, 76) |
| **Diabetes** (%) | 18 | 23 | 20 |
| **Blood loss** (ml)* | 160 (50, 350) | 350 (200, 510) | 190 (100, 300) |
| **OR time** (min)** | 220 (172, 275) | 450 (380, 514) | 406 (353, 458) |
| All values are presented as median (IQR). *p < 0.001 between Total resection and other groups. ** p < 0.001 between all groups | | | |

**Figure S1**

Receiver operating curves for lactate values to predict complications following pancreatic surgery. AUROC for first lactate: 0.531, Highest lactate: 0.581 and postoperative day 1: 0.546


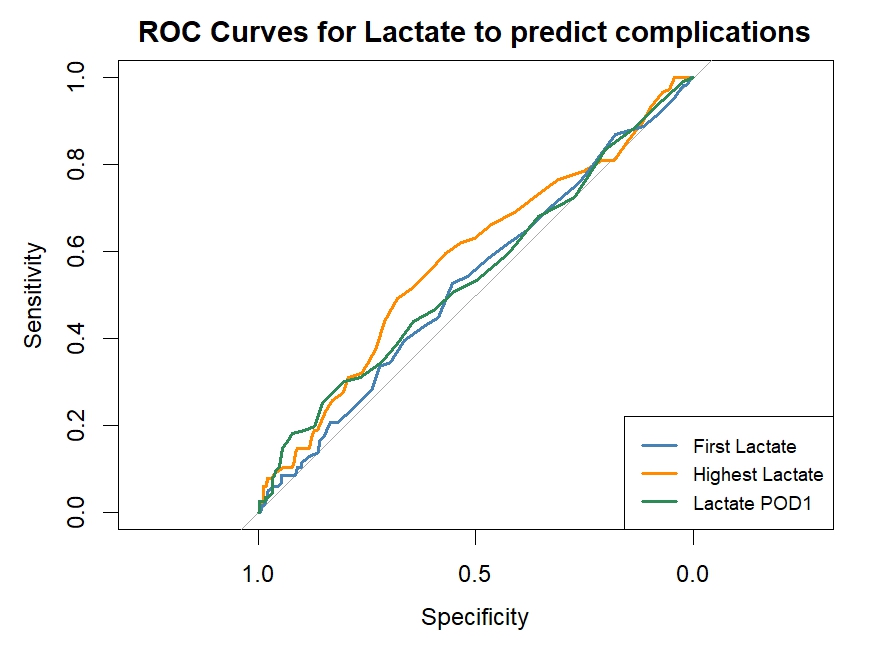


**Figure S2**

Caption: All collected lactate values from arrival at post anesthesia care unit (PACU, t=0) until the first postoperative morning for 491 patients undergoing pancreatic surgery. Data is presented in hourly intervals, total of 2604 observations. Gray lines are patients without postoperative complications and blue lines are patients who suffered complications (Clavien-Dindo ≥ IIIa). Data is presented as median and interquartile range. Linear mixed model indicate that complication group decreases their lactate 0.01 mmol x L^-1^ x h^-1^ slower than the non-complication group (p = 0.165)


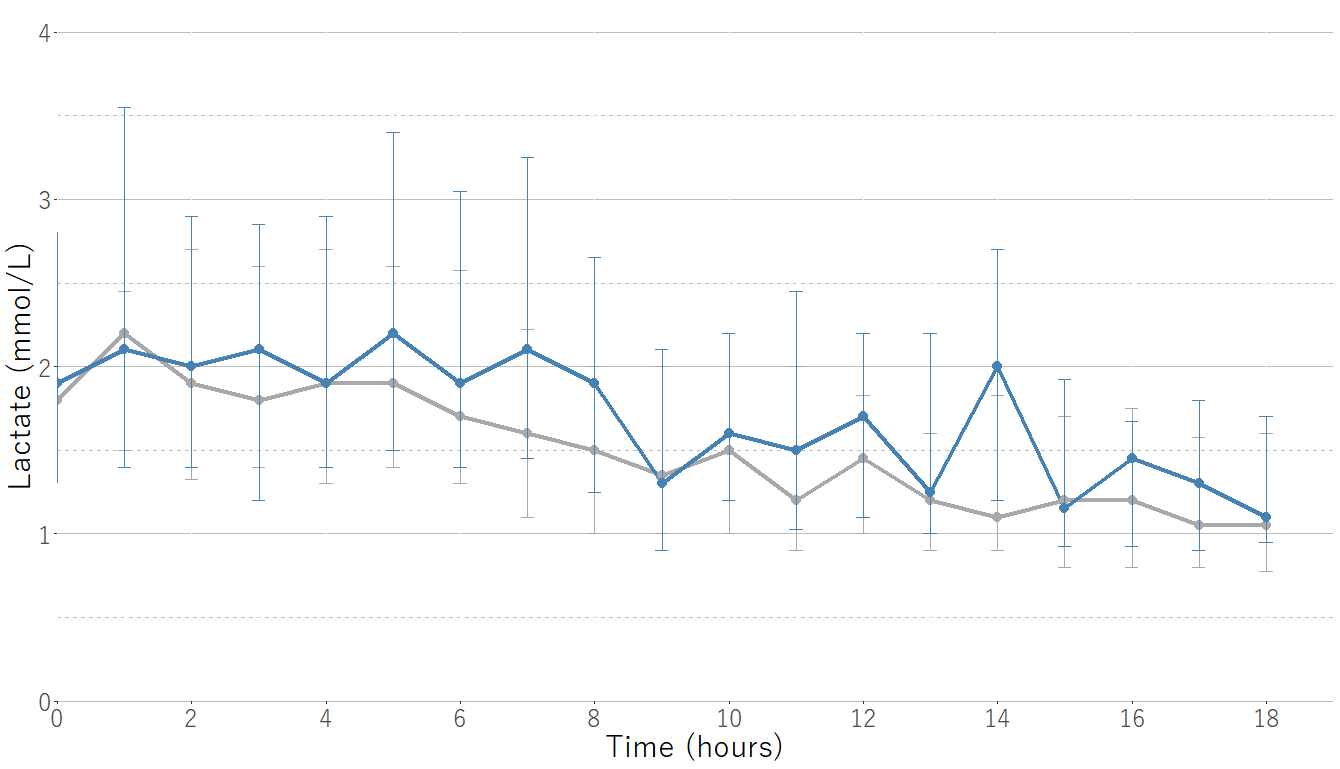

Supplement: Supplementary file 1 [file mmc1.docx]
